# Lottery and Auction on Quantum Blockchain

**DOI:** 10.3390/e22121377

**Published:** 2020-12-05

**Authors:** Xin Sun, Piotr Kulicki, Mirek Sopek

**Affiliations:** 1 Department of the Foundations of Computer Science, John Paul II Catholic University of Lublin, 20-950 Lublin, Poland; xin.sun@kul.pl; 2 MakoLab SA, 91-062 Lodz, Poland; sopek@makolab.com

**Keywords:** quantum blockchain, quantum bit commitment, lottery, auction

## Abstract

This paper proposes a protocol for lottery and a protocol for auction on quantum Blockchain. Our protocol of lottery satisfies randomness, unpredictability, unforgeability, verifiability, decentralization and unconditional security. Our protocol of auction satisfies bid privacy, posterior privacy, bids’ binding, decentralization and unconditional security. Except quantum Blockchain, the main technique involved in both protocols is quantum bit commitment.

## 1. Introduction

A blockchain is a distributed, transparent and append-only ledger of cryptographically linked units of data (blocks). Information is stored in a blockchain in a large decentralized network of parties that do not have to trust one another. The system is distributed in the sense that all nodes of the network, that are in charge of updating the ledger (usually called miners), have separated, identical copies of the ledger. To add a new block to the ledger, the nodes storing the information need to achieve consensus over the content of the ledger.

Cryptocurrencies such as Bitcoin [1] are the best-known applications of blockchain technology. Smart contracts [2], which are enforceable, irrefutable agreements among mutually distrusting peers are another important type of applications. The crucial feature of smart contracts is that they do not require a trusted third party for their administration and enforcement.

Almost all existing blockchain implementations deeply rely on the public-key digital signatures. For that reason, the developments in the field of quantum computing generate a serious threat to them. The factorization tasks, on which the cryptographical power of the public-key digital signatures is based, are hard to solve for traditional computers, but can be easily solved by quantum computers due to quantum algorithms [3]. Quantum computers, discussed for several decades as a theoretical concept, and being in an experimental phase right now, are expected to be ready for wider use quite soon. The current predictions [4] assume that by 2026 the chance of their practical availability is about 15% and by 2031 the chance grows to 50%. As blockchain-based systems are used for the transfer of value, they are particularly vulnerable to an attack. Thus, as pointed out in [5], blockchain technology as we know it today may founder unless it integrates quantum technologies.

There is a significant number of publications related to the quantum-safe blockchain immune to attacks of quantum computers [6,7,8,9,10]. One of the most prominent proposals is the Quantum-secured Blockchain (QB) [6]. It is based on quantum key distribution (QKD) technology that enables an unconditionally secure message authentication. The major limitation of QB is that the consensus protocol it adopts is not efficient, because it becomes exponentially data-intensive if a large number of cheating parties is present. This limitation is overcome in [9], where a new consensus protocol is proposed, with only quadratic dependence of resources on the number of miners.

Quantum-secured Blockchain, as well as other quantum-safe blockchain systems, gives us a general scheme of a distributed ledger, but does not offer protocols for specific tasks like voting, lottery or auction that may be built on top of them. In [10] a simple voting protocol based quantum blockchain is defined. In the present paper, in order to further demonstrate the power and application potential of quantum blockchain, we present protocols for lottery and auction.

While the auction protocol we propose is the first one of the kind, a lottery protocol for quantum blockchain was already mentioned in [9]. The lottery protocol presented there is, however, defined for only two parties and for that reason cannot be applied to the majority of lotteries. In contrast, the protocol we present in the present paper is designed for any number of players and is secured by a group of miners. The main technique that we will use for our lottery and auction protocols, except for quantum blockchain, is quantum bit commitment.

The lottery business is a huge industry of a multi-billion dollar turnover [11]. A lottery is organized by a trusted authority for a usually large number of players. To participate in the game players buy tickets from the organizer. Then, a random process determines the winning tickets. Since revenue is often huge, so is the incentive to cheat. In order to ensure fair play and the trust of players, a lottery protocol should satisfy the following requirements [12,13,14,15,16,17]:

1. Randomness. All tickets are equally likely to win.
2. Unpredictability. No player can predict the winning ticket.
3. Unforgeability. Tickets cannot be forged. Especially, it is impossible to create a winning ticket after the outcome of the random process is known.
4. Verifiablity. The number and the revenue of winning tickets are publicly verifiable.
5. Decentralization. The random process does not rely on a single authority.

Lottery protocols that satisfy the above requirements already exist [12,16]. With the threat from prospective quantum computers, it is reasonable to require that lottery protocols also satisfy another property:

6. Unconditional security. Even an adversary with an unlimited power of computation cannot rig the lottery.

Although quantum coin flipping [18,19,20,21,22,23,24,25], a specific form of lottery, has been researched in the past 20 years, only randomness and the unconditional security has been studied in those works, while other properties of a lottery have rarely been addressed in this context. In this paper, we design a lottery protocol that satisfies all the above requirements appropriate for multiple players.

Auction is an even more important business in the sense that trillions of dollars are transferred by auctions. An auction is a process of buying and selling goods by offering them up for bid, taking bids, and then selling the item to the buyer who offers the highest bid. In general, there are two types of auctions: sealed-bid auction and non-sealed-bid auction. The main advantage of the sealed-bid auction lies in the fact that no buyer gets to know the bids offered by other buyers. In the literature [26,27,28] it is acknowledged that an ideal sealed-bid auction must satisfy the following properties:

1. Bid privacy. The submitted bids are not visible to other buyers during the bidding phase.
2. Posterior privacy. The losing bids are not revealed to the public. In other words, only the seller knows all losing bids and their corresponding buyers.
3. Bids’ binding. Buyers cannot deny or change their bids once they are committed.

In the setting of quantum blockchain, it is reasonable to require that the auction protocol further satisfies the following properties:

4. Decentralization. The process of the auction does not rely on a single trusted third party.
5. Unconditional security. Even an adversary with an unlimited power of computation cannot manipulate the process of auction.

While blockchain-based auction [29,30] does satisfy decentralization and quantum auction [31,32] does satisfy unconditional security, no existing auction protocol satisfies both of these properties. The auction protocol we are going to propose satisfies all the above properties.

The rest of the paper is organized as follows. In Section 2 we review some background knowledge of quantum blockchain and quantum bit commitment. In Section 3 we present our lottery protocol and in Section 4 our auction protocol. In Section 5 we present conclusions and remarks on the future work.

## 2. Background

### 2.1. Quantum Blockchain

The concept of quantum blockchain was presented in [6,9,10]. We are using this general framework to specify lottery and auction protocols. We assume that each pair of nodes is connected by a quantum channel and a classical channel. Every pair of nodes can establish a sequence of secret keys by using the quantum key distribution [33] mechanisms. Those keys will later be used for secure communication.

New transactions or new messages (updates) on the blockchain are initiated by the nodes that wish to append some new data to the chain. Each miner checks the consistency of the update with respect to their local copy of the ledger and works out a judgment regarding the update’s admissibility. Then, all the miners apply a consensus algorithm to the update, arriving at a consensus regarding the correct version of the update.

In this paper, we will consider quantum blockchain on a high level, omitting its detailed structure and mechanism, and taking advantage of its following desired properties:

1. Every node is a (small scale) quantum computer which can run some quantum computation on a small number of qubits. More specifically, nodes are capable of performing the quantum computation involved in at least one quantum bit commitment protocol.
2. The communication between different nodes is unconditionally secure.
3. There is a consensus algorithm which can be used by all miners to achieve consensus. The consensus mechanism is immune to attacks. A general definition of the consensus algorithm is given as the following.

**Definition** **1** (consensus algorithm)**.** *An algorithm among n parties, in which every party p holds an input value $x_{p}\in D$ (for some finite domain D) and eventually, decide on an output value in $y_{p}\in D$, is said to achieve consensus if the algorithm guarantees that the output values of all honest parties are the same.*

### 2.2. Quantum Bit Commitment

Bit commitment typically consists of two phases, namely: commitment and opening. In the commitment phase, a sender chooses a bit *a* (a=0 or 1) and presents to a receiver some evidence about it. In the opening phase, the sender discloses more information to the receiver. That information enables the receiver to reconstruct the initial bit. Let us use $a^{\prime }$ to call the reconstructed bit. A useful bit commitment should be correct, concealing and binding. A correct bit commitment protocol will ensure that the initial bit is equal to the reconstructed one: $a=a^{\prime }$. A protocol is concealing if a receiver cannot get to know the bit before the opening phase, and is binding if a sender cannot change the bit after the commitment phase.

The design of the first quantum bit commitment (QBC) protocol can be attributed to Bennett and Brassard [33]. A number of QBC protocols have been designed to achieve unconditional security (see e.g., [34,35]). Although according to the Mayers-Lo-Chau (MLC) no-go theorem [36,37,38], unconditionally secure QBC cannot be achieved within the theory of quantum mechanics, scientists have found ways to overcome this negative result in the past two decades. Among them, let us mention cheat-sensitive quantum bit commitment (CSQBC) [39,40,41,42,43] and relativistic QBC [44,45,46,47,48,49] protocols. Accompanied by well-designed punishment mechanisms the CSQBC can be useful in practice and resilient to the attack of quantum computers. Relativistic QBC protocols make use of relativity theory and also achieve unconditional security (see [49], where a protocol is presented in which a bit is concealed for 24 hours). Another practically useful QBC can be found in He [50,51], who proposed a QBC protocol based on the use of Mach-Zehnder interferometer.

The following is an abstract yet rigorous definition of QBC, which can be found in Sun et al. [38] and will be used in this paper.

**Definition** **2** (quantum bit commitment)**.** *A quantum bit commitment protocol consists of the following:*

(1) *Two finite-dimensional Hilbert spaces A and B.*
(2) *A function commit:{0,1}↦A⊗B.*
(3) *Two pure states $|c_{0}\rangle ,|c_{1}\rangle \in A⊗B$, in which $|c_{i}\rangle =commit\left(i\right)$ is the commitment of i.*
(4) *A quantum operation (i.e., completely positive, trace-preserving super operator) Open on A⊗B such that $Open(|c_{0}\rangle \langle c_{0}|)\neq Open(|c_{1}\rangle \langle c_{1}|)$.*

*This QBC protocol is concealing if $Tr_{A}(|c_{0}\rangle \langle c_{0}|)=Tr_{A}(|c_{1}\rangle \langle c_{1}|)$. It is binding if there is no unitary U on A such that $\left(U⊗I_{B}\right)|c_{0}\rangle =|c_{1}\rangle$.*

## 3. Lottery on Quantum Blockchain

**Figure 1:**
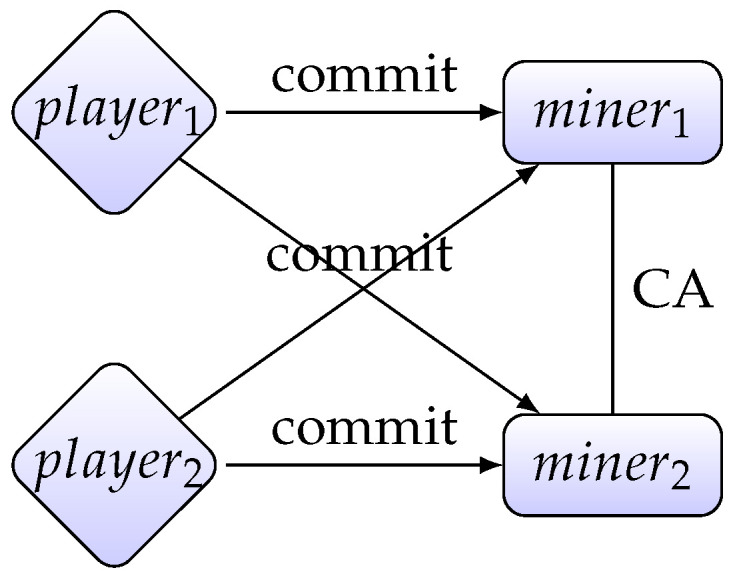
A network of players and miners: players commit their tickets to miners. Miners use a consensus algorithm (CA) to achieve consensus about the players’ tickets.

Now let us present our lottery protocol. In the setting of the lottery, we assume there are *n* players and every ticket of the lottery is an *m*-bit string. Our lottery protocol consists of three phases: the ticket purchasing phase, the ticket agreement phase and the winner determination phase. Figure 1 presents a simplified visualization of our protocol.

1. Ticket purchasing:
  (a) For every player $p_{i}\in \left\{p_{1},\ldots ,p_{n}\right\}$, to purchase a ticket $T_{i}$, $p_{i}$ uses QBC to commit $T_{i}$ to all miners. At the end of this phase, every miner possesses a list of commitments $\left(commit\left(T_{1}\right),\ldots ,commit\left(T_{n}\right)\right)$.
2. Ticket agreement:
  (a) Every player opens his commitment to every miner, so that the commitments in every miner’s possession change to $(Open\left(commit\left(T_{1}\right)\right),\ldots ,$$Open(commit\left(T_{n}\right)))$, which essentially equals to $\left(T_{1},\ldots ,T_{n}\right)$.
  (b) All the miners run a consensus algorithm to achieve a consensus on the tickets $\left(T_{1},\ldots ,T_{n}\right)$ purchased by players. Every miner adds $\left(T_{1},\ldots ,T_{n}\right)$ to his local copy of the blockchain.
3. Winner determination:
  (a) The winning ticket is calculated by bit-wise XOR: $T=T_{1}⊕\ldots ⊕T_{n}$.
  (b) A player’s revenue is determined by the Hamming distance between his ticket and the winning ticket *T*. The closer his ticket is to the winning ticket, the higher is his revenue (a specific rule of revenue which satisfies this principle is beyond the scope of this paper and is left for future work).

### Analysis

Our lottery protocol satisfies the following requirements:

1. **Randomness**.
  The winning ticket is calculated by bit-wise XOR. For every index j∈{1,…,m} in the winning ticket, T[j]=1 iff $T_{1}\left[j\right]⊕\ldots ⊕T_{n}\left[j\right]=1$. Therefore, the probability of T[j]=1 is the same as T[j]=0.
2. **Unpredictability**.
  To predict the winning ticket a player has to know all tickets before they are opened. The concealing property of QBC ensures that even miners cannot know the players’ tickets before they are opened. Since tickets are only sent to the miners by QBC, the probability that a player knows all tickets is even lower than the probability that a miner knows them.
3. **Unforgeability**.
  The binding property of QBC ensures that it is impossible to change a ticket after the ticket purchasing phase.
4. **Verifiablity**.
  This is because the quantum blockchain is a transparent database. After the ticket agreement phase, the list $\left(T_{1},\ldots T_{n}\right)$ is added to the blockchain. Every player can read all the other players’ tickets and calculate the winning ticket by himself.
5. **Decentralization**.
  The random process does not rely on a single authority. Every player’s ticket essentially affects the calculation of the winning ticket.Moreover, the calculation of the winning ticket does not rely on a single miner, but on all miners.
6. **Unconditional security**.
  Even an adversary with an unlimited power of computation cannot manipulate the lottery protocol. The concealing and binding property of QBC does not rely on any computational assumption. Nor does the security of the consensus algorithm. The unconditional security of the ledger is further guaranteed by the unconditional security of the digital signature schemes adopted by quantum Blockchain.

## 4. Auction on Quantum Blockchain

**Figure 2:**
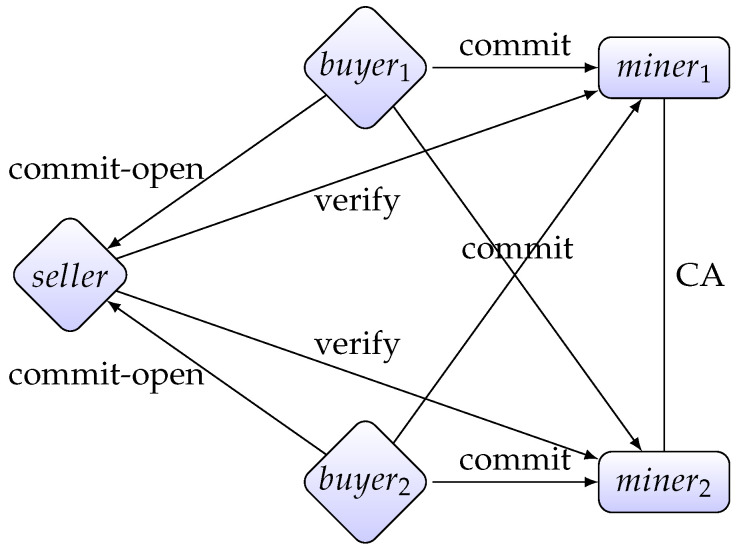
A network of the seller, buyers and miners.

In our protocol of auction, we assume three types of participants: one seller *S*, *m* buyers $\left\{B_{1},\ldots ,B_{m}\right\}$ and *n* miners $\left\{M_{1},\ldots ,M_{n}\right\}$. Our protocol works as follows: First all buyers send their bids to the seller. Then the seller calculates which buyer is the winner. Finally, all miners verify the seller’s calculation. Figure 2 is a brief visualization of the process of auction. There are five phases in our protocol.

1. The bidding phase: Every buyer $B_{i}$ commits his bid $b_{i}$ to the seller and to all miners $M_{j}$, where $b_{i}$ is a positive integer.
2. The opening phase: Every buyer opens his bid to the seller.
3. Decision phase: The seller calculates the winning bid, which is the highest bid (if there is a tie, then one of the maximal bids is chosen randomly), and the winning buyer, who has offered the winning bid.
4. Verification phase: In this phase, the seller *S* and every miner $M_{j}$ (1≤j≤n) run the following procedure to convince $M_{j}$ that *S* has chosen the valid winner:
  (a) *S* sends the information about the winning buyer $B_{w}$ and his bid $b_{w}$ to the miner $M_{j}$.
  (b) *S* permutes losing bids to obtain a new list of m−1 bids $\left(b_{1}^{\prime },\ldots ,b_{m-1}^{\prime }\right)$.
  (c) *S* sends $b_{1}^{\prime },\ldots ,b_{m-1}^{\prime }$ to $M_{j}$.
  (d) $M_{j}$ first checks if $b_{w}\geq b_{k}^{\prime }$ for all k∈{1,…,m−1}. If yes, then $M_{j}$ sends $\left(b_{w},b_{1}^{\prime },\ldots ,b_{m-1}^{\prime }\right)$ to all buyers. Otherwise, $M_{j}$ sets *S* as a cheater by setting output to ⊥.
  (e) After receiving $\left(b_{w},b_{1}^{\prime },\ldots ,b_{m-1}^{\prime }\right)$, every buyer $B_{i}$ checks if his bid is in the list, i.e., there is some $b_{k}^{\prime }=b_{i}$. If yes, then $B_{i}$ sends the message “valid” to $M_{j}$. Otherwise $B_{i}$ opens $b_{i}$ to $M_{j}$. $M_{j}$ then sets *S* as a cheater by setting output to ⊥.
  (f) If $M_{j}$ does not output ⊥, then the seller passes the verification phase. The output of $M_{j}$ is now $\left(b_{w},b_{1}^{\prime },\ldots ,b_{m-1}^{\prime },B_{w}\right)$
5. Publication phase: All miners run the consensus algorithm to achieve consensus on the output of the verification phase. The consensus is then added to the blockchain.

### Analysis

Our auction protocol satisfies the following requirements:

1. **Bid privacy**.
  Every buyer only commits and opens his bids to the seller. Therefore, no buyer knows other buyers’ bids.
2. **Posterior privacy**.
  What is added to the blockchain is the winning buyer and his bid, as well as a permuted list of losing bids. Therefore, no losing buyer’s bid is revealed.
3. **Bids’ binding**.
  The binding property of quantum bit commitment ensures that buyers cannot deny or change their bids once they are committed.
4. **Decentralization**.
  There are in total *n* miners. The process of the auction does not rely on a single miner.
5. **Unconditional security**.
  As in the case of our lottery protocol, even an adversary with an unlimited power of computation cannot manipulate the auction protocol because the security of the quantum bit commitment and consensus algorithm does not depend on computational complexity. The unconditional security of the ledger relies on quantum Blockchain properties.

## 5. Conclusions and Future Work

This paper proposes a lottery protocol and an auction protocol based on quantum bit commitment and quantum blockchain. These protocols satisfy all the important properties of distributed lottery/auction and are implementable by the current technology.

In the future, we are interested in applying quantum blockchain to the general field of multi-party computation. We believe that quantum blockchain will provide new insights into these interesting tasks. We estimate that in the future more complicated protocols (smart contracts) on the quantum blockchain will be designed. Developing a formal tool for the specification and verification of smart contracts on the quantum blockchain is on our agenda. The recently developed categorical logic of quantum programs [52] seems to be a good starting point.
